# The genome sequence of *Tenthredo notha *Klug, 1814, a sawfly

**DOI:** 10.12688/wellcomeopenres.17811.2

**Published:** 2024-03-28

**Authors:** Steven Falk, University of Oxford and Wytham Woods Genome Acquisition Lab, Darwin Tree of Life Barcoding collective, Wellcome Sanger Institute Tree of Life programme, Wellcome Sanger Institute Scientific Operations: DNA Pipelines collective, Tree of Life Core Informatics collective, Gavin R. Broad, Darwin Tree of Life Consortium

**Affiliations:** 1 Independent Researcher, Kenilworth, Warwickshire, UK; 2 Department of Life Sciences, Natural History Museum, London, UK

**Keywords:** Tenthredo notha, sawfly, genome sequence, chromosomal, Hymenoptera

## Abstract

We present a genome assembly from an individual female *Tenthredo notha* (Arthropoda; Insecta; Hymenoptera; Tenthredinidae). The genome sequence is 253 megabases in span. Most of the assembly (99.91%) is scaffolded into 20 chromosomal pseudomolecules. The mitochondrial genome was also assembled and is 19.8 kilobases in length. Gene annotation of this assembly on Ensembl has identified 10,235 protein coding genes.

## Species taxonomy

Eukaryota; Metazoa; Ecdysozoa; Arthropoda; Hexapoda; Insecta; Pterygota; Neoptera; Endopterygota; Hymenoptera; Tenthredinoidea; Tenthredinidae; Tenthredininae; Tenthredo; *Tenthredo notha* (Klug, 1814) (NCBI:txid362091).

## Background

*Tenthredo notha* is a yellow- and black-striped sawfly, one of a group of several similar wasp-mimicking species. It can be separated from close relatives by details of the yellow markings on the abdomen and the black apex to the hind tibia ( Benson, 1952; Fekete, 2018), although males can be tricky to identify. The green larvae (with a pale lateral stripe) feed on white clover ( *Trifolium repens*) and tufted vetch ( *Vicia cracca*); like the food plants, *T. notha* is widely distributed across Britain, although under-recorded. *Tenthredo notha* is found throughout Central, Northern and South-East Europe and Eastwards widely through Russia ( Taeger, 1988) and China ( Wei *et al.*, 2006).

Flying mainly in July and August, the flight time helps distinguish *T. notha* from the very similar *Tenthredo arcuata* Forster, 1771, which is typically a spring flyer. As with other *Tenthredo* species, adults can often be found on flowers, particularly of Apiaceae.

As the first chromosome-level genome for the subfamily Tenthredininae, this will help with research into the diversification of this large group of often conspicuous sawflies, a particularly North temperate radiation. Previous limited sequence data for *Tenthredo notha* has been used in a study of tenthredinid phylogeny, concentrating on the large subfamily Nematinae ( Nyman *et al.,* 2006). There is still much work to do in reconstructing the phylogeny of Tenthredininae.

## Genome sequence report

**Figure 1.**
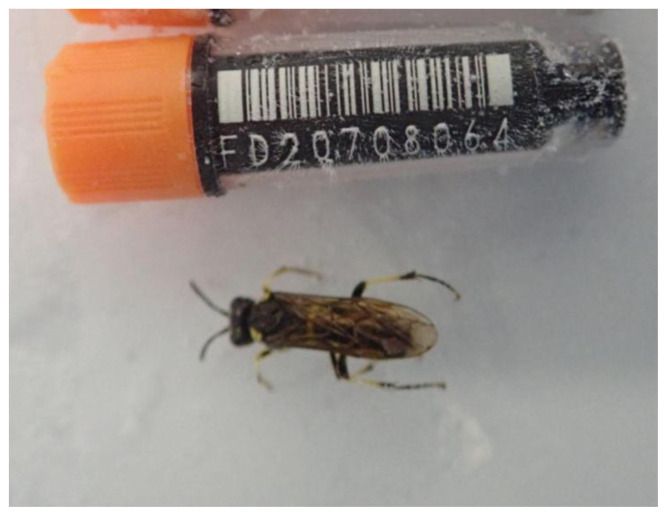
Image of the *Tenthredo notha* (iyTenNoth1) specimen taken during preservation and processing.

The genome was sequenced from a single *T. notha* ( Figure 1) collected from Wytham Woods, Oxfordshire (biological vice-county: Berkshire), UK (latitude 51.769, longitude -1.339). A total of 77-fold coverage in Pacific Biosciences single-molecule long reads and 262-fold coverage in 10X Genomics read clouds were generated. Primary assembly contigs were scaffolded with chromosome conformation Hi-C data. Manual assembly curation corrected 91 missing/misjoins and removed 29 haplotypic duplications, reducing the assembly size by 4.90% and the scaffold number by 70.79%, and increasing the scaffold N50 by 22.36%.

**Figure 2.**
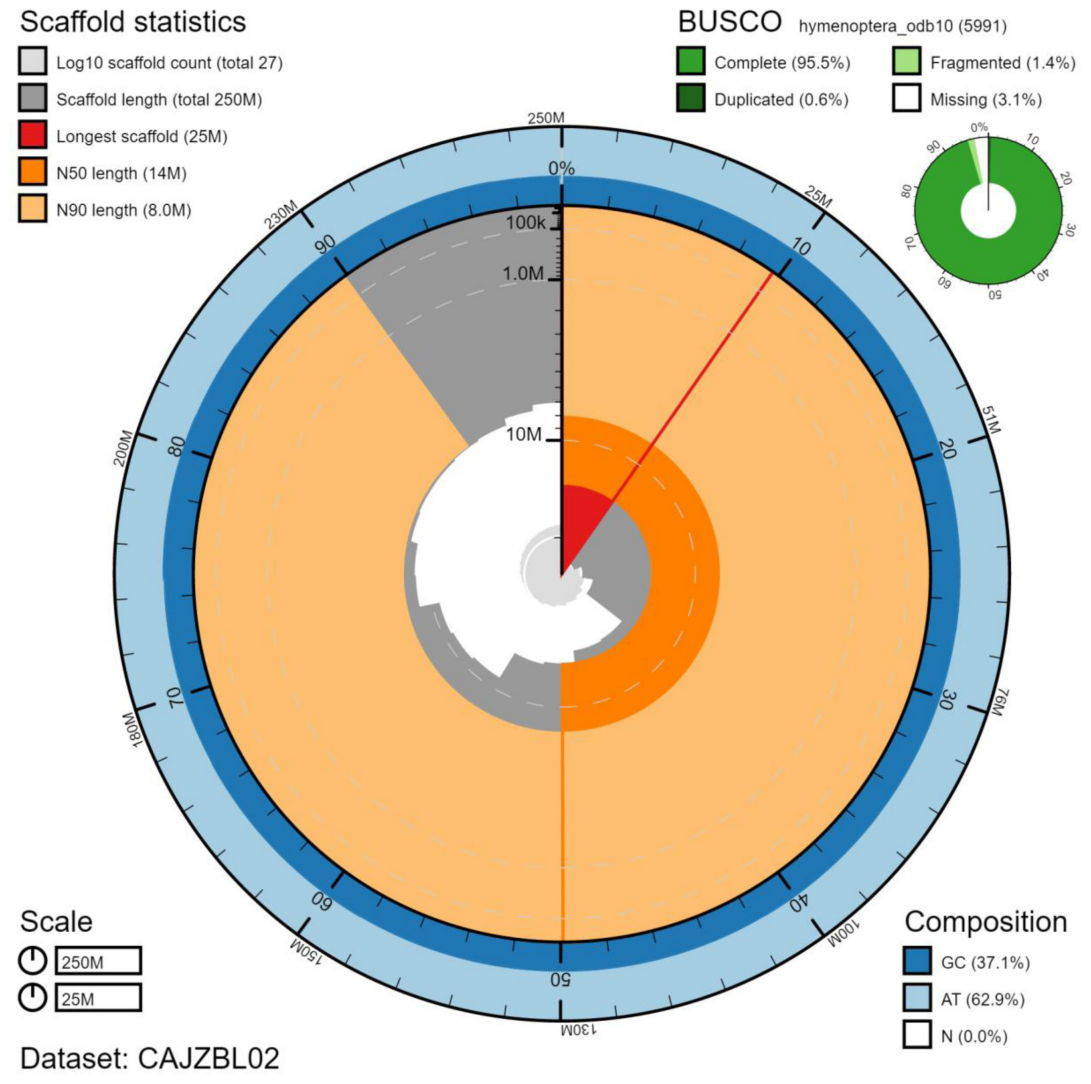
Genome assembly of *Tenthredo notha*, iyTenNoth1.2: metrics. The BlobToolKit Snailplot shows N50 metrics and BUSCO gene completeness. The main plot is divided into 1,000 size-ordered bins around the circumference with each bin representing 0.1% of the 252,838,151 bp assembly. The distribution of chromosome lengths is shown in dark grey with the plot radius scaled to the longest chromosome present in the assembly (24,575,054 bp, shown in red). Orange and pale-orange arcs show the N50 and N90 chromosome lengths (13,966,764 and 8,019,486 bp), respectively. The pale grey spiral shows the cumulative chromosome count on a log scale with white scale lines showing successive orders of magnitude. The blue and pale-blue area around the outside of the plot shows the distribution of GC, AT and N percentages in the same bins as the inner plot. A summary of complete, fragmented, duplicated and missing BUSCO genes in the hymenoptera_odb10 set is shown in the top right. An interactive version of this figure is available at https://blobtoolkit.genomehubs.org/view/iyTenNoth1.2/dataset/CAJZBL02/snail.

The final assembly has a total length of 253 Mb in 26 sequence scaffolds with a scaffold N50 of 14.0 Mb ( Table 1). Of the assembly sequence, 99.91% was assigned to 20 chromosomal-level scaffolds (numbered by sequence length) ( Figure 2– Figure 5; Table 2). The assembly has a BUSCO v5.2.2 ( Manni *et al.,* 2021) completeness of 95.5% (single 94.9%, duplicated 0.6%) using the hymenoptera_odb10 reference set (n=5,991).

The assembly is that of a diploid female. While not fully phased, the assembly deposited is of one haplotype. Contigs corresponding to the second haplotype have also been deposited.

Metadata for specimens, barcode results, spectra estimates, sequencing runs, contaminants and pre-curation assembly statistics are given at https://links.tol.sanger.ac.uk/species/362091.

**Table 1.** Genome data for *Tenthredo notha*, iyTenNoth1.2.

*Project accession data*	
Assembly identifier	iyTenNoth1.2
Species	*Tenthredo notha*
Specimen	Specimen ID Ox000858 TOLID iyTenNoth1
NCBI taxonomy ID	NCBI:txid362091
BioProject	PRJEB46306
BioSample ID	SAMEA7746761
Isolate information	Thorax (genome assembly), head (Hi-C), abdomen (RNA sequencing)
*Raw data accessions*	
PacificBiosciences SEQUEL II	ERR6939232
10X Genomics Illumina	ERR6688454-ERR6688457
Hi-C Illumina	ERR6688458
RNA-Seq Illumina	ERR9434994
*Genome assembly*	
Assembly accession	GCA_914767705.2
*Accession of alternate haplotype*	GCA_914767975.1
Span (Mb)	253
Number of contigs	97
Contig N50 length (Mb)	7.0
Number of scaffolds	26
Scaffold N50 length (Mb)	14.0
Longest scaffold (Mb)	24.6
BUSCO * genome score	C:95.5%[S:94.9%,D:0.6%],F:1.4%, M:3.1%,n:5,991

*BUSCO scores based on the hymenoptera_odb10 BUSCO set using v5.2.2. C= complete [S= single copy, D=duplicated], F=fragmented, M=missing, n=number of orthologues in comparison. A full set of BUSCO scores is available at https://blobtoolkit.genomehubs.org/view/iyTenNoth1.2/dataset/CAJZBL02/busco.

**Figure 3.**
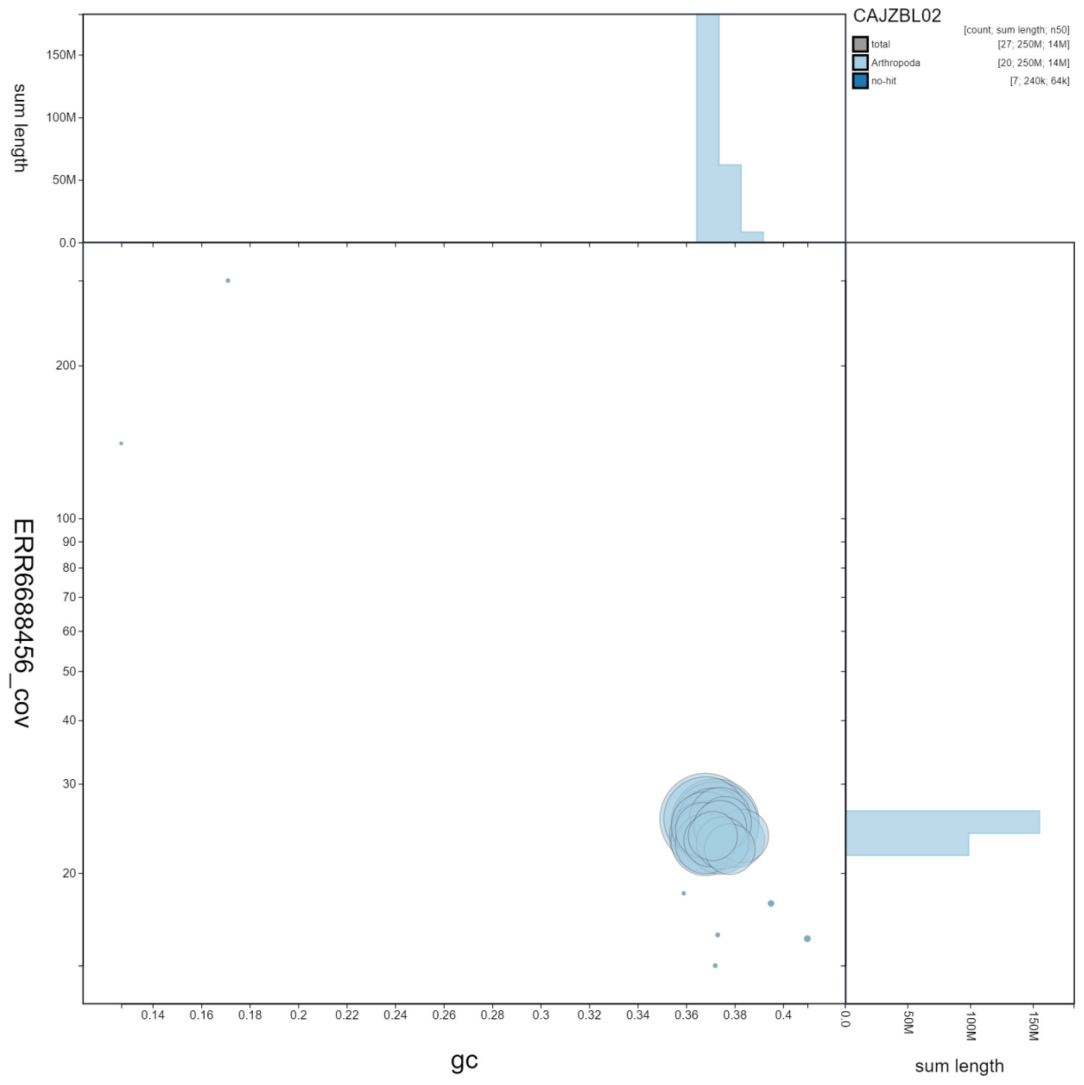
Genome assembly of *Tenthredo notha*, iyTenNoth1.2. GC coverage. BlobToolKit GC-coverage plot. Scaffolds are coloured by phylum. Circles are sized in proportion to scaffold length. Histograms show the distribution of scaffold length sum along each axis. An interactive version of this figure is available at https://blobtoolkit.genomehubs.org/view/iyTenNoth1.2/dataset/CAJZBL02/blob.

**Figure 4.**
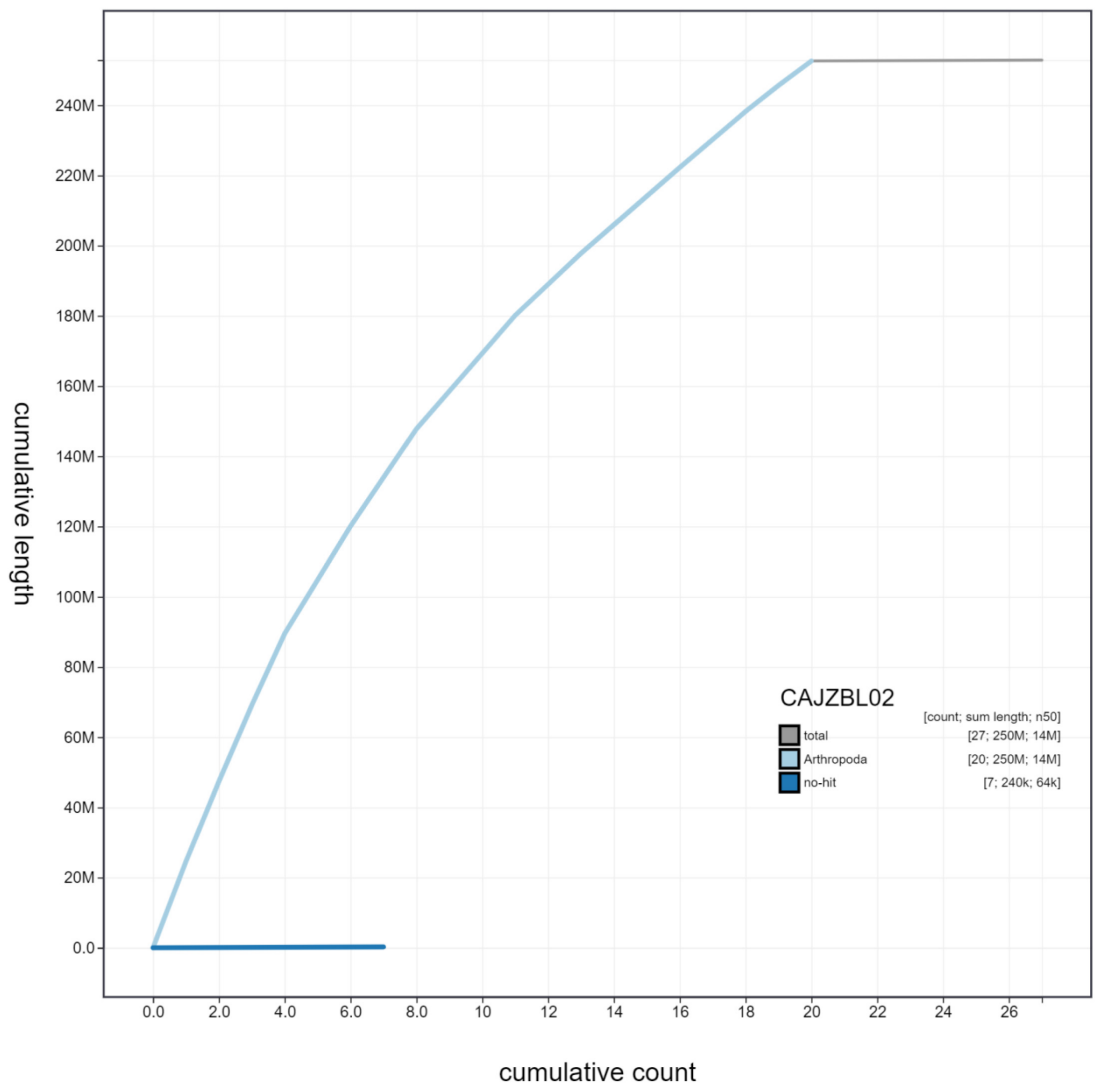
Genome assembly of *Tenthredo notha*, iyTenNoth1.2: cumulative sequence. BlobToolKit cumulative sequence plot. The grey line shows cumulative length for all scaffolds. Coloured lines show cumulative lengths of scaffolds assigned to each phylum using the buscogenes taxrule. An interactive version of this figure is available at https://blobtoolkit.genomehubs.org/view/iyTenNoth1.2/dataset/CAJZBL02/cumulative.

**Figure 5.**
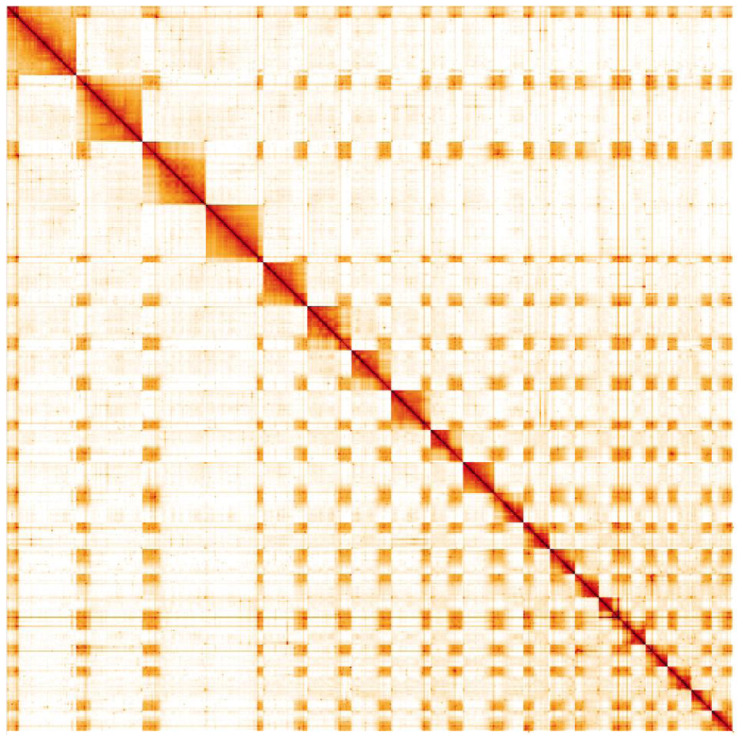
Genome assembly of *Tenthredo notha*, iyTenNoth1.2: Hi-C contact map. Hi-C contact map of the iyTenNoth1.2 assembly, visualised in HiGlass. Chromosomes are shown in size order from left to right and top to bottom. The interactive Hi-C map can be viewed here.

**Table 2.** Chromosomal pseudomolecules in the genome assembly of *Tenthredo notha*, iyTenNoth1.2.

INSDC accession	Chromosome	Size (Mb)	GC%
OU611906.1	1	24.58	36.8
OU611907.1	2	22.78	37.2
OU611908.1	3	21.79	37.2
OU611909.1	4	20.43	36.8
OU611910.1	5	15.34	37.1
OU611911.1	6	15.20	36.8
OU611912.1	7	13.97	36.8
OU611913.1	8	13.72	36.8
OU611914.1	9	10.99	37.4
OU611915.1	10	10.77	37.4
OU611916.1	11	10.58	36.8
OU611917.1	12	8.88	36.7
OU611918.1	13	8.79	38.1
OU611919.1	14	8.23	38.3
OU611920.1	15	8.14	37.6
OU611921.1	16	8.12	37.0
OU611922.1	17	8.02	37.4
OU611923.1	18	7.91	37.5
OU611924.1	19	7.39	37.8
OU611925.1	20	6.97	37.1
OU611926.1	MT	0.02	17.2
-	Unplaced	0.22	37.9

## Genome annotation report

The iyTenNoth1.1 genome has been annotated using the Ensembl rapid annotation pipeline ( Table 1; https://rapid.ensembl.org/Tenthredo_notha_GCA_914767705.1/Info/Index). The resulting annotation includes 16,290 transcribed mRNAs from 10,235 protein-coding and 1,484 non-coding genes. There are 1.44 coding transcripts per gene and 6.62 exons per transcript.

## Methods

### Sample acquisition and nucleic acid extraction

A female *T. notha* (specimen ID Ox000858, ToLID iyTenNoth1) was collected from Wytham Woods, Oxfordshire (biological vice-county: Berkshire), UK (latitude 51.769, longitude -1.339) from woodland by Steven Falk, Independent Researcher, using a net. The sample was formally identified by the same individual and snap-frozen on dry ice.

DNA was extracted at the Tree of Life laboratory, Wellcome Sanger Institute. The iyTenNoth1 sample was weighed and dissected on dry ice with tissue set aside for Hi-C sequencing. Thorax tissue was disrupted using a Nippi Powermasher fitted with a BioMasher pestle. Fragment size analysis of 0.01–0.5 ng of DNA was then performed using an Agilent FemtoPulse. High molecular weight (HMW) DNA was extracted using the Qiagen MagAttract HMW DNA extraction kit. Low molecular weight DNA was removed from a 200-ng aliquot of extracted DNA using 0.8X AMpure XP purification kit prior to 10X Chromium sequencing; a minimum of 50 ng DNA was submitted for 10X sequencing. HMW DNA was sheared into an average fragment size between 12-20 kb in a Megaruptor 3 system with speed setting 30. Sheared DNA was purified by solid-phase reversible immobilisation using AMPure PB beads with a 1.8X ratio of beads to sample to remove the shorter fragments and concentrate the DNA sample. The concentration of the sheared and purified DNA was assessed using a Nanodrop spectrophotometer and Qubit Fluorometer and Qubit dsDNA High Sensitivity Assay kit. Fragment size distribution was evaluated by running the sample on the FemtoPulse system.

RNA was extracted from abdomen tissue of iyTenNoth1 in the Tree of Life Laboratory at the WSI using the RNA Extraction: Automated MagMax™ *mir*Vana protocol ( do Amaral *et al.*, 2023). The RNA concentration was assessed using a Nanodrop spectrophotometer and a Qubit Fluorometer using the Qubit RNA Broad-Range Assay kit. Analysis of the integrity of the RNA was done using the Agilent RNA 6000 Pico Kit and Eukaryotic Total RNA assay.

Protocols developed by the WSI Tree of Life laboratory are publicly available on protocols.io ( Denton *et al.*, 2023). 

### Sequencing

Pacific Biosciences HiFi circular consensus and 10X Genomics read cloud sequencing libraries were constructed according to the manufacturers’ instructions. DNA sequencing was performed by the Scientific Operations core at the Wellcome Sanger Institute on Pacific Biosciences SEQUEL II and Illumina NovaSeq 6000 instruments. RNA sequence data was generated on an Illumina HiSeq 4000 instrument. Hi-C data were generated from head tissue of iyTenNoth1 using the Arima v2.0 kit and sequenced on an Illumina NovaSeq 6000 instrument.

### Genome assembly

Assembly was carried out with Hifiasm ( Cheng *et al.,* 2021); haplotypic duplication was identified and removed with purge_dups ( Guan *et al.,* 2020). One round of polishing was performed by aligning 10X Genomics read data to the assembly with longranger align, calling variants with freebayes ( Garrison & Marth, 2012). The assembly was then scaffolded with Hi-C data ( Rao *et al.,* 2014) using SALSA2 ( Ghurye *et al.,* 2019). The assembly was checked for contamination as described previously ( Howe *et al.,* 2021). Manual curation ( Howe *et al.,* 2021) was performed using HiGlass ( Kerpedjiev *et al.,* 2018) and Pretext. The mitochondrial genome was assembled using MitoHiFi ( Uliano-Silva *et al.,* 2021), which performed annotation using MitoFinder ( Allio *et al.,* 2020). The genome was analysed and BUSCO scores generated within the BlobToolKit environment ( Challis *et al.,* 2020). Table 3 contains a list of all software tool versions used, where appropriate.

**Table 3.** Software tools used.

Software tool	Version	Source
Hifiasm	0.15.1	Cheng *et al.*, 2021
purge_dups	1.2.3	Guan *et al.*, 2020
SALSA2	2.2	Ghurye *et al.*, 2019
longranger align	2.2.2	https://support.10xgenomics.com/ genome-exome/software/pipelines/ latest/advanced/other-pipelines
freebayes	v1.3.1-17-gaa2ace8	Garrison & Marth, 2012
MitoHiFi	2	https://github.com/marcelauliano/ MitoHiFi
HiGlass	1.11.6	Kerpedjiev *et al.*, 2018
PretextView	0.2.x	https://github.com/wtsi-hpag/ PretextView
BlobToolKit	3.0.5	Challis *et al.*, 2020

### Genome annotation

The Ensembl gene annotation system ( Aken *et al.,* 2016) was used to generate annotation for the Tenthredo notha assembly ( GCA_914767705.1). Annotation was created primarily through alignment of transcriptomic data to the genome, with gap filling via protein-to-genome alignments of a select set of proteins from UniProt ( UniProt Consortium, 2019).

### Ethics/compliance issues

The materials that have contributed to this genome note have been supplied by a Darwin Tree of Life Partner. The submission of materials by a Darwin Tree of Life Partner is subject to the Darwin Tree of Life Project Sampling Code of Practice. By agreeing with and signing up to the Sampling Code of Practice, the Darwin Tree of Life Partner agrees they will meet the legal and ethical requirements and standards set out within this document in respect of all samples acquired for, and supplied to, the Darwin Tree of Life Project. Each transfer of samples is further undertaken according to a Research Collaboration Agreement or Material Transfer Agreement entered into by the Darwin Tree of Life Partner, Genome Research Limited (operating as the Wellcome Sanger Institute), and in some circumstances other Darwin Tree of Life collaborators.

## Data Availability

European Nucleotide Archive: Tenthredo notha. Accession number PRJEB46306; https://identifiers.org/ena.embl/PRJEB46306. The genome sequence is released openly for reuse. The *T. notha* genome sequencing initiative is part of the Darwin Tree of Life (DToL) project. All raw sequence data and the assembly have been deposited in INSDC databases. Raw data and assembly accession identifiers are reported in Table 1.

## Author information

Members of the University of Oxford and Wytham Woods Genome Acquisition Lab are listed here: https://doi.org/10.5281/zenodo.5746938.

Members of the Darwin Tree of Life Barcoding collective are listed here: https://doi.org/10.5281/zenodo.5744972.

Members of the Wellcome Sanger Institute Tree of Life programme are listed here: https://doi.org/10.5281/zenodo.6125027.

Members of Wellcome Sanger Institute Scientific Operations: DNA Pipelines collective are listed here: https://doi.org/10.5281/zenodo.5746904.

Members of the Tree of Life Core Informatics collective are listed here: https://doi.org/10.5281/zenodo.6125046.

Members of the Darwin Tree of Life Consortium are listed here: https://doi.org/10.5281/zenodo.5638618.

## References

[ref-1] Aken BL Ayling S Barrell D : The Ensembl Gene Annotation System. *Database (Oxford).* 2016;2016: baw093. 10.1093/database/baw093 27337980 PMC4919035

[ref-2] Allio R Schomaker-Bastos A Romiguier J : MitoFinder: Efficient Automated Large-Scale Extraction of Mitogenomic Data in Target Enrichment Phylogenomics. *Mol Ecol Resour.* 2020;20(4):892–905. 10.1111/1755-0998.13160 32243090 PMC7497042

[ref-3] Benson RB : Hymenoptera 2, Symphyta, Section (b). In: *Handbooks for the Identification of British Insects*.1952;6. Reference Source

[ref-4] Challis R Richards E Rajan J : BlobToolKit – Interactive Quality Assessment of Genome Assemblies. *G3 (Bethesda).* 2020;10(4):1361–1374. 10.1534/g3.119.400908 32071071 PMC7144090

[ref-5] Cheng H Concepcion GT Feng X : Haplotype-Resolved *de Novo* Assembly Using Phased Assembly Graphs with Hifiasm. *Nat Methods.* 2021;18(2):170–75. 10.1038/s41592-020-01056-5 33526886 PMC7961889

[ref-19] Denton A Yatsenko H Jay J : Sanger Tree of Life Wet Laboratory Protocol Collection V.1. *protocols.io*.2023. 10.17504/protocols.io.8epv5xxy6g1b/v1

[ref-20] do Amaral RJV Bates A Denton A : Sanger Tree of Life RNA Extraction: Automated MagMax ^TM^ mirVana. *protocols.io*.2023. 10.17504/protocols.io.6qpvr36n3vmk/v1

[ref-6] Fekete K : Beginner’s Guide to Identifying British Tenthredo. Natural History Museum, London,2018. Reference Source

[ref-7] Garrison E Marth G : Haplotype-Based Variant Detection from Short-Read Sequencing. arXiv: 1207.3907.2012. 10.48550/arXiv.1207.3907

[ref-8] Ghurye J Rhie A Walenz BP : Integrating Hi-C Links with Assembly Graphs for Chromosome-Scale Assembly. *PLoS Comput Biol.* 2019;15(8): e1007273. 10.1371/journal.pcbi.1007273 31433799 PMC6719893

[ref-9] Guan D McCarthy SA Wood J : Identifying and Removing Haplotypic Duplication in Primary Genome Assemblies. *Bioinformatics.* 2020;36(9):2896–2898. 10.1093/bioinformatics/btaa025 31971576 PMC7203741

[ref-10] Howe K Chow W Collins J : Significantly Improving the Quality of Genome Assemblies through Curation. *GigaScience.* 2021;10(1): giaa153. 10.1093/gigascience/giaa153 33420778 PMC7794651

[ref-11] Kerpedjiev P Abdennur N Lekschas F : HiGlass: Web-Based Visual Exploration and Analysis of Genome Interaction Maps. *Genome Biol.* 2018;19(1): 125. 10.1186/s13059-018-1486-1 30143029 PMC6109259

[ref-12] Manni M Berkeley MR Seppey M : BUSCO Update: Novel and Streamlined Workflows along with Broader and Deeper Phylogenetic Coverage for Scoring of Eukaryotic, Prokaryotic, and Viral Genomes. *Mol Biol Evol.* 2021;38(10):4647–4654. 10.1093/molbev/msab199 34320186 PMC8476166

[ref-13] Nyman T Zinovjev AG Vikberg V : Molecular Phylogeny of the Sawfly Subfamily Nematinae (Hymenoptera: Tenthredinidae). *Syst Entomol.* 2006;31(4):569–83. 10.1111/j.1365-3113.2006.00336.x

[ref-14] Rao SSP Huntley MH Durand NC : A 3D Map of the Human Genome at Kilobase Resolution Reveals Principles of Chromatin Looping. *Cell.* 2014;159(7):1665–80. 10.1016/j.cell.2014.11.021 25497547 PMC5635824

[ref-15] Taeger A : Dritter Beitrag Zur Kenntnis Der Blattwespengattung *Tenthredo* L.(Hymenoptera: Symphyta: Tenthredinidae). *Beitr Ent.* 1988;38(2):337–359. Reference Source

[ref-16] Uliano-Silva M Nunes JGF Krasheninnikova K : marcelauliano/MitoHiFi: mitohifi_v2.0.2021. 10.5281/zenodo.5205678

[ref-17] UniProt Consortium: UniProt: A Worldwide Hub of Protein Knowledge. *Nucleic Acids Res.* 2019;47(D1):D506–15. 10.1093/nar/gky1049 30395287 PMC6323992

[ref-18] Wei M Nie H Taeger A : Sawflies (Hymenoptera: Symphyta) of China-Checklist and Review of Research. *Recent Sawfly Research: Synthesis and Prospects.* Goecke & Evers, Keltern,2006;16:505–74. Reference Source

