# Supplementary material for: The genome sequence of  Tenthredo notha Klug, 1814, a sawfly
Source: Wellcome Open Res. 2024 Mar 28;7:120. Originally published 2022 Apr 4. [Version 2] doi: 10.12688/wellcomeopenres.17811.2 (PMC10997988; doi:10.12688/wellcomeopenres.17811.2)
Supplement: Peer review history

# Reviewer report: Reviewer response for version 2

**DOI:** 10.21956/wellcomeopenres.23604.r78219

**Published:** 2024-04-05

**Authors:** Saskia Wutke

**Affiliations:** 1 Department of Environmental and Biological Sciences, University or Eastern Finland, Joensuu, Finland

Thank you for incorporating the suggested changes into the report.

Are sufficient details of methods and materials provided to allow replication by others?

Partly

Is the rationale for creating the dataset(s) clearly described?

Yes

Are the datasets clearly presented in a useable and accessible format?

Yes

Are the protocols appropriate and is the work technically sound?

Yes

Reviewer Expertise:

insect genomics and phylogenetics

I confirm that I have read this submission and believe that I have an appropriate level of expertise to confirm that it is of an acceptable scientific standard.

# Reviewer report: Reviewer response for version 2

**DOI:** 10.21956/wellcomeopenres.23604.r78220

**Published:** 2024-04-01

**Authors:** Marko Prous

**Affiliations:** 1 Museum of Natural History, University of Tartu, Tartu, Estonia

Thank you for the corrections. I have no further comments.

Are sufficient details of methods and materials provided to allow replication by others?

Yes

Is the rationale for creating the dataset(s) clearly described?

Yes

Are the datasets clearly presented in a useable and accessible format?

Yes

Are the protocols appropriate and is the work technically sound?

Yes

Reviewer Expertise:

Systematics of sawflies (Hymenoptera).

I confirm that I have read this submission and believe that I have an appropriate level of expertise to confirm that it is of an acceptable scientific standard.

# Reviewer report: Reviewer response for version 1

**DOI:** 10.21956/wellcomeopenres.19719.r50552

**Published:** 2022-05-31

**Authors:** Saskia Wutke

**Affiliations:** 1 Department of Environmental and Biological Sciences, University or Eastern Finland, Joensuu, Finland

This data note describes the sequencing, assembly and annotation of the genome for Tenthredo notha, a sawfly in the family Tenthredinidae, using Pacbio, 10X linked read and Hi-C sequencing. Overall, the dataset is nicely presented and the methods are state-of-the-art and of high quality. Yet, I would wish for a few more details concerning the sample (I was wondering why the sex is unknown) and genome annotation. It is stated that transcriptomic data was used for annotating the genome, but the origin/source of this data is not provided i.e., the transcriptome of which taxa was used? 

 Additionally, the background section could be more detailed and include also some taxonomic placement to support its relevance.

Are sufficient details of methods and materials provided to allow replication by others?

Partly

Is the rationale for creating the dataset(s) clearly described?

Yes

Are the datasets clearly presented in a useable and accessible format?

Yes

Are the protocols appropriate and is the work technically sound?

Yes

Reviewer Expertise:

insect genomics and phylogenetics

I confirm that I have read this submission and believe that I have an appropriate level of expertise to confirm that it is of an acceptable scientific standard, however I have significant reservations, as outlined above.

# Reviewer report: Reviewer response for version 1

**DOI:** 10.21956/wellcomeopenres.19719.r50276

**Published:** 2022-05-11

**Authors:** Marko Prous

**Affiliations:** 1 Museum of Natural History, University of Tartu, Tartu, Estonia

I don't have anything to say about the genome data. It's a valuable resource and seems to be of good quality (I have already used it to fish out some gene sequences used for other sawflies).

 My main concern is poor documentation of the specimen used for sequencing.

 Strangely the sex of the specimen is not known. Knowing the sex is important for species identification, so how is it possible that the identifier did not know the sex? It seems that the whole specimen was not destroyed for DNA extraction, then it should be trivial to report the sex (if tip of abdomen is still preserved).

 If the specimen is preserved, in which collection is it stored? What is the specimen voucher code/specimen ID (Ox000858 or iyTenNoth1?) and is the specimen labelled accordingly to unambiguously associate the specimen with the sequence data?

 In the abstract correct "Halictidae" to Tenthredinidae.

 In table 1 clarify specimen number. In GenBank the specimen ID is Ox000858 and iyTenNoth1 is 'tolid' (don't know what is the purpose of this number).

Are sufficient details of methods and materials provided to allow replication by others?

Yes

Is the rationale for creating the dataset(s) clearly described?

Yes

Are the datasets clearly presented in a useable and accessible format?

Yes

Are the protocols appropriate and is the work technically sound?

Yes

Reviewer Expertise:

Systematics of sawflies (Hymenoptera).

I confirm that I have read this submission and believe that I have an appropriate level of expertise to confirm that it is of an acceptable scientific standard, however I have significant reservations, as outlined above.
